# Machine learning for prediction of postoperative nausea and vomiting in patients with intravenous patient-controlled analgesia

**DOI:** 10.1371/journal.pone.0277957

**Published:** 2022-12-22

**Authors:** Jae-Geum Shim, Kyoung-Ho Ryu, Eun-Ah Cho, Jin Hee Ahn, Yun Byeong Cha, Goeun Lim, Sung Hyun Lee

**Affiliations:** 1 Department of Anesthesiology and Pain Medicine, College of Medicine, Graduate School, Kyung Hee University, Seoul, Korea; 2 Department of Anesthesiology and Pain Medicine, Kangbuk Samsung Hospital, Sungkyunkwan University School of Medicine, Seoul, Korea; Hanyang University, REPUBLIC OF KOREA

## Abstract

**Background:**

Postoperative nausea and vomiting (PONV) is a still highly relevant problem and is known to be a distressing side effect in patients. The aim of this study was to develop a machine learning model to predict PONV up to 24 h with fentanyl-based intravenous patient-controlled analgesia (IV-PCA).

**Methods:**

From July 2019 and July 2020, data from 2,149 patients who received fentanyl-based IV-PCA for analgesia after non-cardiac surgery under general anesthesia were applied to develop predictive models. The rates of PONV at 1 day after surgery were measured according to patient characteristics as well as anesthetic, surgical, or PCA-related factors. All statistical analyses and computations were performed using the R software.

**Results:**

A total of 2,149 patients were enrolled in this study, 337 of whom (15.7%) experienced PONV. After applying the machine-learning algorithm and Apfel model to the test dataset to predict PONV, we found that the area under the receiver operating characteristic curve using logistic regression was 0.576 (95% confidence interval [CI], 0.520–0.633), k-nearest neighbor was 0.597 (95% CI, 0.537–0.656), decision tree was 0.561 (95% CI, 0.498–0.625), random forest was 0.610 (95% CI, 0.552–0.668), gradient boosting machine was 0.580 (95% CI, 0.520–0.639), support vector machine was 0.649 (95% CI, 0.592–0.707), artificial neural network was 0.686 (95% CI, 0.630–0.742), and Apfel model was 0.643 (95% CI, 0.596–0.690).

**Conclusions:**

We developed and validated machine learning models for predicting PONV in the first 24 h. The machine learning model showed better performance than the Apfel model in predicting PONV.

## Background

Postoperative nausea and vomiting (PONV) is a common condition and is known to be a distressing side effect in patients [1]. The incidence of PONV is 30% and can be as high as 80% in high-risk patients [2, 3]. Although the mechanism of PONV is not clear, the use of perioperative opioids is known to be associated with it [4]. Nonetheless, opioid-based intravenous patient-controlled analgesia (IV-PCA) currently plays an important role in routine postoperative analgesic therapy [5–7]. Therefore, by accurately predicting PONV, patients can be warned of the risk of developing PONV, and clinicians can be assisted in making decisions about preventive treatment.

Apfel’s risk score is a simple assessment tool derived to predict the 24-h rates of PONV [8, 9]. However, the Apfel model does not guarantee the accurate prediction of the risk of PONV, with limited discrimination and calibration properties [10, 11]. Recently, studies have used dynamic predictive models or machine learning to improve the predictive performance of PONV [12–15].

Machine learning is the application of artificial intelligence, whereby a computer algorithm automatically learns and improves from prior experience [16]. The machine learning algorithm produces an inferred function that can be used as the predictor of new data after sufficient training with known input and output values [17]. It may be used for prediction in the medical field. Recently, machine learning algorithms have shown high performance in various fields of medicine, such as diagnosis, prognosis, and clinical decision support [18–21].

To the best of our knowledge, no previous study has compared the performance of Apfel and machine learning methods in predicting PONV 24 h after surgery. We expect our research results to improve the prediction of PONV and quality of patient care.

## Methods and methods

### Study population

We collected data from patients (>19 years) after non-cardiac surgery under general anesthesia who received fentanyl-based IV-PCA at Kangbuk Samsung Hospital between July 2019 and July 2020. The exclusion criteria for this study were refusal to receive PCA and admission to the intensive care unit. This study was reviewed and approved by the Institutional Review Board (IRB No. 2020-08-001) of Kangbuk Samsung Hospital (Seoul, Korea). This study was conducted in accordance with the principles of the Declaration of Helsinki of the World Medical Association. The need for written informed consent was waived as this was a retrospective study of electronic medical records.

### Data collection

The rates of PONV at 1 day after surgery were measured with information on postoperative pain scores and other complications by the PCA team in our hospital. We also included patient characteristics as well as anesthetic, surgical, or PCA-related factors in the predictive models. The continuous variables were age, body mass index (BMI), duration of anesthesia, and dosage of fentanyl in IV-PCA. The categorical variables were sex, history of motion sickness or PONV, American Society of Anesthesiologists (ASA) physical status, diabetes mellitus, hypertension, premedication, use of preintubation opioids, anesthetic agents (sevoflurane, desflurane, or TIVA), intraoperative remifentanil infusion, the use of intraoperative opioids (fentanyl or meperidine), emergency operation, laparoscopic surgery, type of surgery, adjuvant nefopam, and antiemetic (ramosetron) in IV-PCA. Continuous variables were transformed to values between 0 and 1 by minimum-maximum normalization, implemented in the caret package in the R software.

### Feature selection

Feature selection is the process of selecting features that contribute the most to our prediction variable, leading to improved performance. In this process, recursive feature elimination was used as a method that fits the random forest function in the core of the model and removes the weakest feature until the specified number of features is reached. Features are ranked by the model’s feature importance by iteratively eliminating a small number of features per loop. To enable the machine learning algorithms to run efficiently, we only used the data features resulting from recursive feature elimination to train our machine learning models.

### Model assessment

To determine the goodness of the prediction ability, model performance was evaluated by comparing machine learning approaches to the Apfel model in terms of the area under the receiver operating characteristic curve (AUROC). The AUROC was plotted using the test dataset to understand the tradeoff in performance for different threshold values in imbalanced classification problems. We also compared the accuracy, sensitivity, and specificity.

The confusion matrix is used for summarizing the performance of a classification problem as shown in Table 1. Accuracy, sensitivity, and specificity are described in terms of true positive (TP), true negative (TN), false negative (FN) and false positive (FP).

**Table 1:** Confusion matrix.

		Actual	
		Positive	Negative
Predicted	Positive	TP	TN
	Negative	FP	FN

TP, true positive; TN, true negative; FN, false negative; FP, false positive

The accuracy of model is the ratio of correct predictions to total predictions made and is defined as

Accuracy = (TN + TP) / (TN+TP+FN+FP)

The sensitivity of model is the proportion of actual positive cases that are correctly identified and is defined as

Sensitivity = TP / (TP + FN)

The specificity of model is the proportion of actual negative cases that are correctly identified and is defined as

Specificity = TN / (TN + FP)

### Statistical analysis

All statistical analyses and computations were performed using the R software version 3.6.3 (R Development Core Team, Vienna, Austria). The machine learning algorithm was implemented using the following packages: Caret (https://CRAN.R-project.org/package=caret), Xgboost (https://CRAN.R-project.org/package=xgboost), and Keras (https://CRAN.R-project.org/package=keras). The entire code of our study (https://github.com/jgshim/PONV) is provided.

Before applying the machine learning models, our data set was randomly divided into 70/30 training and test sets, as we did not want our models to overfit and generalize well. Specifically, 70% of the data was used for training prediction models, and 30% was used as the testing set for verification. A 10-fold cross-validation repeated three times was used to assess how the predictive model generalizes to an independent dataset. The missing data were imputed using the nearest neighbor imputation algorithms, where each missing value is replaced by a value obtained from related cases in the entire data set [22]. The synthetic minority oversampling technique method, addressing imbalanced classification problems, was used to oversample the minority class and balance the low incidence of PONV in the training set [23].

## Results

### Patient’s characteristics

**Fig 1:**
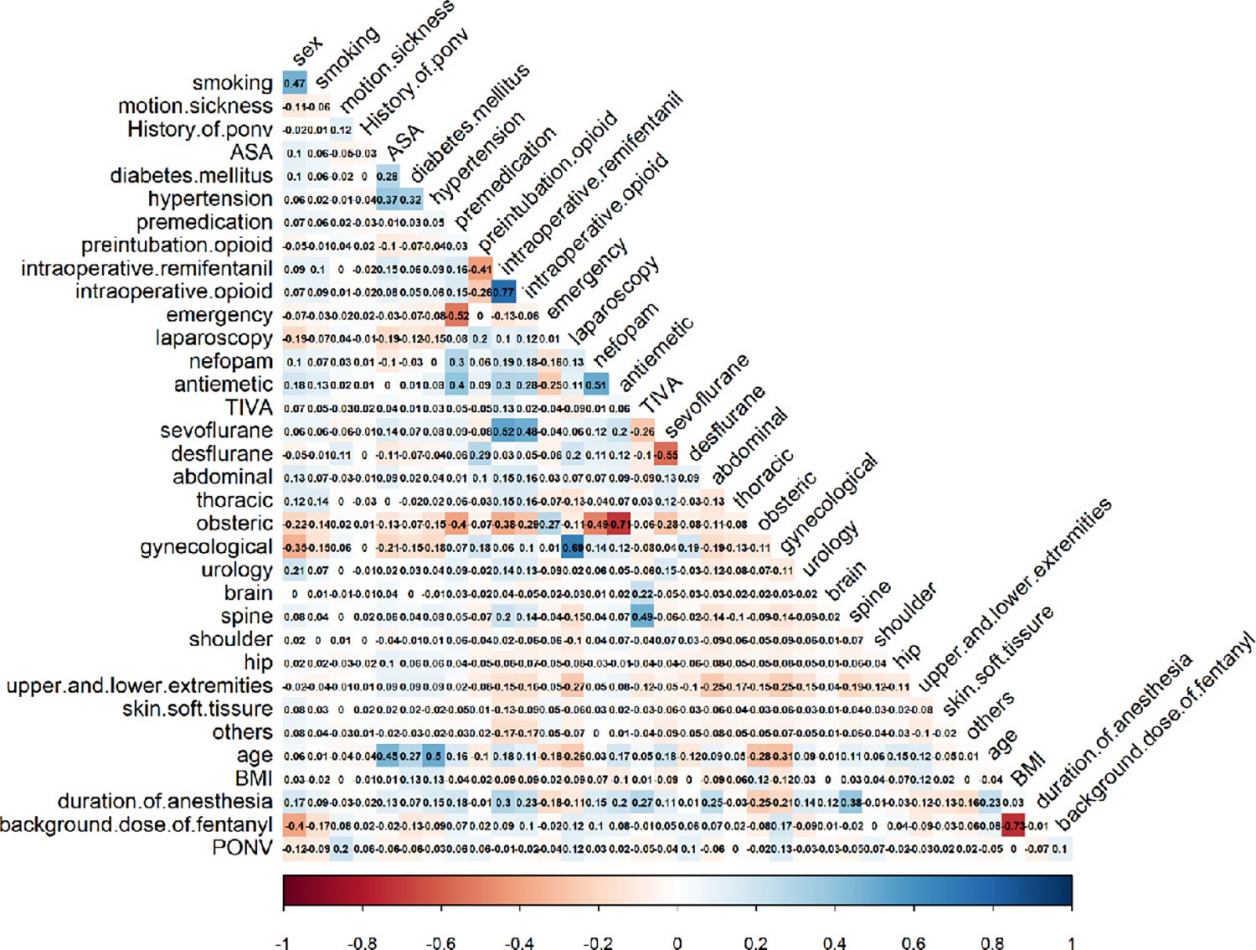
Correlation between variables.

The sample group included 2,680 patients who received fentanyl-based IV-PCA for analgesia after non-cardiac surgery under general anesthesia at Kangbuk Samsung Hospital between July 2019 and July 2020. A total of 23 patients aged ≤18 years were excluded. In addition, 508 patients were excluded because they were subjected to regional anesthesia. As a result, a total of 2,149 patients satisfying all inclusion criteria were enrolled in the study. During the 24 h after surgery, 337 patients (15.7%) experienced PONV. The patient characteristics as well as anesthetic, surgical, or PCA-related variables are summarized in Table 2. The correlation analysis showed a weak positive correlation between motion sickness, laparoscopy, desflurane, and gynecology surgery and PONV, as shown in Fig 1. However, male sex and smoking status showed a weak negative correlation with PONV.

**Table 2:** Dataset characteristics.

	All cases	No PONV	PONV	*P*-value
	(N = 2,149)	(*n* = 1,812)	(*n* = 337)	
**Patient characteristics**				
Age (y)	60 (47–71)	61 (48–71)	57 (43–68)	< 0.001
Sex (female)	1,248 (58.1%)	997 (55.0%)	251 (74.5%)	< 0.001
BMI (kg/m^2^)	24.3 (22.2–26.7)	24.3 (22.2–26.7)	24.2 (22.1–26.8)	0.44
History of smoking	523 (24.3%)	477 (26.3%)	46 (13.6%)	< 0.001
History of motion sickness	133 (5.0%)	64 (3.5%)	49 (14.5%)	< 0.001
History of PONV	16 (0.7%)	8 (0.4%)	8 (2.4%)	< 0.001
ASA physical status				< 0.001
ASA I	504 (23.5%)	401 (22.1%)	103 (30.6%)	
ASA II	1,013 (47.1%)	847 (46.7%)	166 (49.3%)	
ASA III	632 (29.4%)	564 (31.1%)	68 (20.2%)	
Diabetes mellitus	421 (19.6%)	373 (20.6%)	48 (14.2%)	0.009
Hypertension	794 (36.9%)	690 (38.1%)	104 (30.9%)	0.01
**Anesthetic factor**				
Duration of anesthesia (min)	160 (115–220)	165 (115–225)	145 (105–195)	< 0.001
Premedication (anticholinergic)	1,809 (84.2%)	1,510 (83.3%)	299 (88.7%)	0.02
Preintubation opioid	254 (11.8%)	197 (10.9%)	57 (16.9%)	0.002
Anesthetics (%) in G/A				< 0.001
Sevoflurane	1,547 (72.0%)	1,328 (73.3%)	219 (65.0%)	
Desflurane	479 (22.3%)	369 (20.4%)	110 (32.6%)	
TIVA	123 (5.7%)	115 (6.3%)	8 (2.4%)	
Intraoperative remifentanil infusion (%)	1,879 (87.4%)	1,597 (88.1%)	282 (83.7%)	0.03
Intraoperative opioids (fentanyl, meperidine)	1,788 (83.2%)	1,524 (84.1%)	264 (78.3%)	0.01
**Surgical factor**				
Emergency (%)	199 (9.3%)	180 (9.9%)	19 (5.6%)	0.02
Laparoscopic surgery	465 (21.6%)	352 (19.4%)	113 (33.5%)	< 0.001
Type of surgery				< 0.001
Abdominal	420 (19.5%)	376 (20.8%)	44 (13.1%)	
Thoracic	213 (9.9%)	181 (10.0%)	32 (9.5%)	
Obstetric	20 (0.9%)	16 (0.9%)	4 (1.2%)	
Gynecological	401 (18.7%)	295 (16.3%)	106 (31.5%)	
Urology	174 (8.1%)	154 (8.5%)	20 (5.9%)	
Brain	10 (0.5%)	10 (0.6%)	0 (0.0%)	
Spine	262 (12.2%)	237 (13.1%)	25 (7.4%)	
Shoulder	111 (5.2%)	80 (4.4%)	31 (9.2%)	
Hip	44 (2.0%)	39 (2.2%)	5 (1.5%)	
Upper and lower extremities	444 (20.7%)	382 (21.1%)	62 (18.4%)	
Skin, soft tissue	20 (0.9%)	17 (0.9%)	3 (0.9%)	
Others	30 (1.4%)	25 (1.4%)	5 (1.5%)	
**PCA-related factor**				
Background dose of fentanyl in PCA (μg/kg/h)	0.356 (0.315–0.407)	0.353 (0.313–0.404)	0.370 (0.328–0.424)	< 0.001
Adjuvant nefopam in PCA	1,909 (88.8%)	1,606 (88.6%)	303 (89.9%)	0.55
Antiemetic, Ramosetron	2,077 (96.6%)	1,752 (96.7%)	325 (96.4%)	0.94

BMI, body mass index; ASA, American Society of Anesthesiologists; TIVA, total intravenous anesthesia; PCA, patient-controlled analgesia

### Feature selection

We identified 21 variables, including patient characteristics as well as anesthetic, surgical, or PCA-related factors, from previous studies conducted to identify features that may contribute to PONV. Among these variables, anesthetics and type of surgery were categorical variables with more than two levels. As an input for our models, categorical variables with n levels were transformed into n variables, each with two levels. As a result, 34 variables were initially considered as input variables for the model.

**Fig 2:**
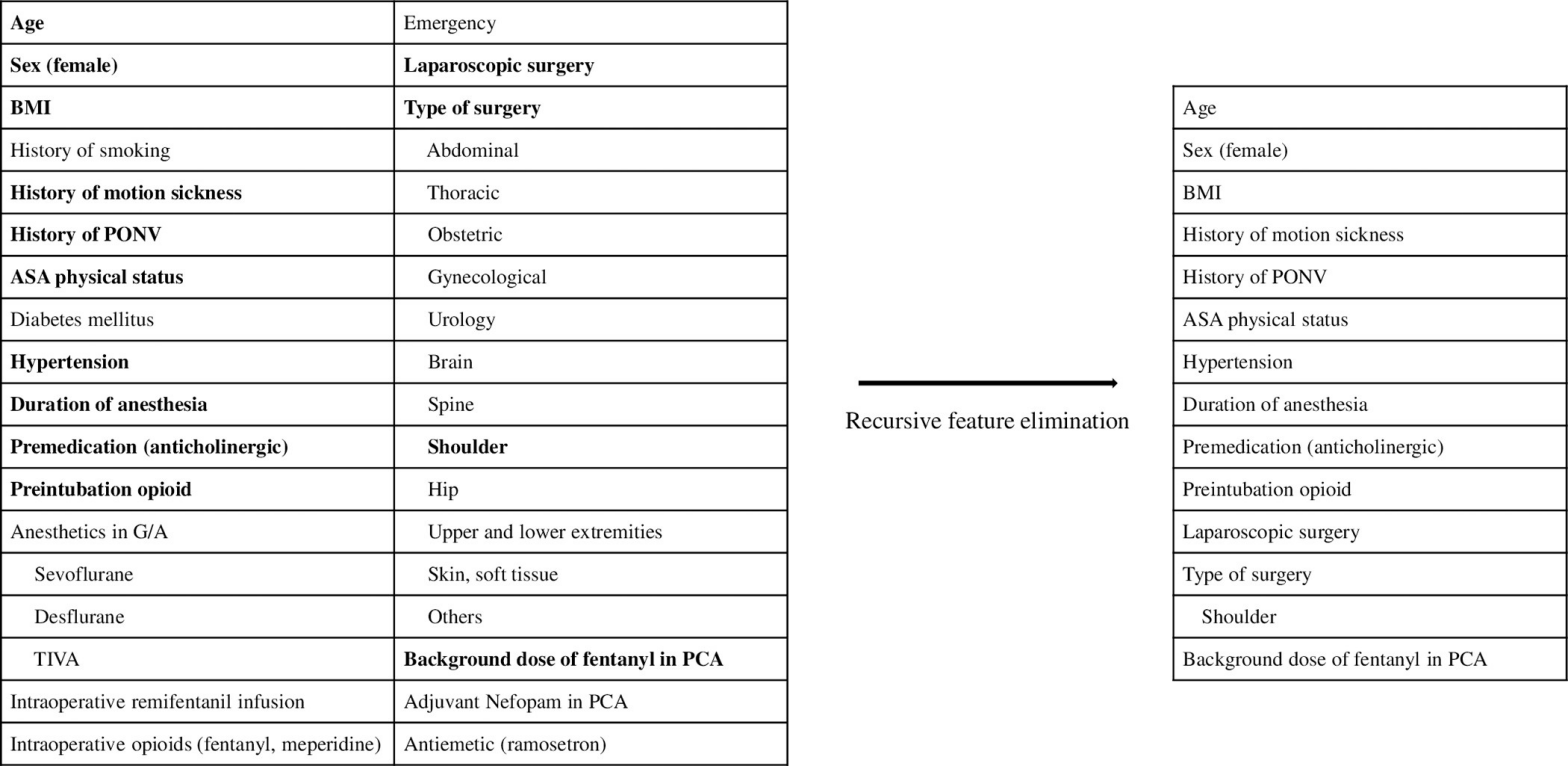
Feature selection process by recursive feature elimination on the training dataset.

The recursive feature elimination algorithm resulted in the final 13 factors contributing to PONV. Fig 2 shows the process of feature selection after the step of recursive feature elimination. On final feature selection, only 13 features were used as input variables in training the machine learning models for predicting PONV.

### Model performance

**Fig 3:**
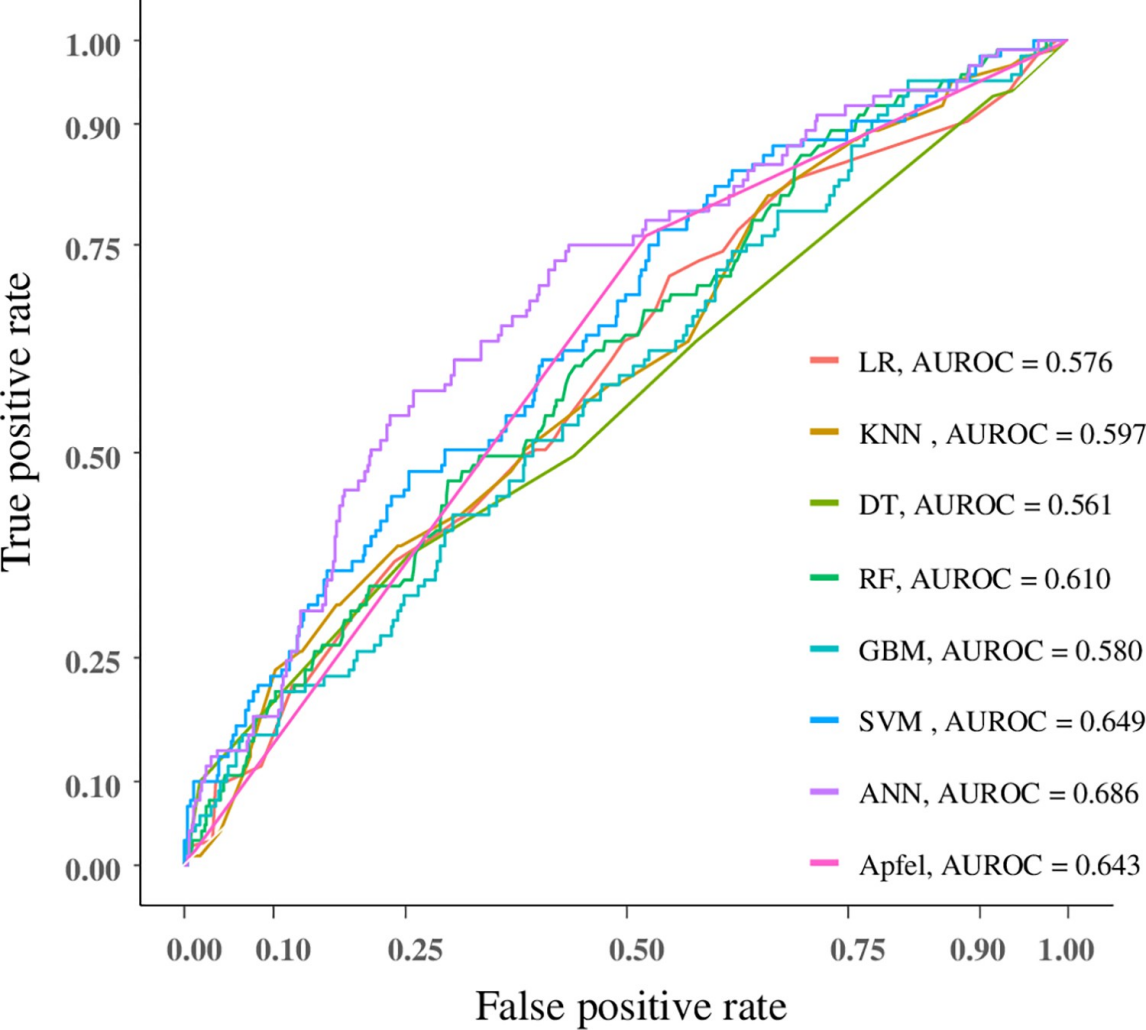
Areas under the receiver operating curve for the machine learning and Apfel model.

The predictive performance of various machine learning and Apfel models is shown in Table 3. After applying the test dataset for all machine learning techniques and the Apfel score to predict PONV, we found that the AUROC using logistic regression was 0.576 (95% confidence interval [CI], 0.520–0.633), k-nearest neighbor was 0.597 (95% CI, 0.537–0.656), decision tree was 0.561 (95% CI, 0.498–0.625), random forest was 0.610 (95% CI, 0.552–0.668), gradient boosting machine was 0.580 (95% CI, 0.520–0.639), support vector machine (SVM) was 0.649 (95% CI, 0.592–0.707), artificial neural network (ANN) was 0.686 (95% CI, 0.630–0.742), and Apfel score was 0.643 (95% CI, 0.596–0.690). The ANN showed the largest AUROC (0.686, 95% CI, 0.630–0.742), as shown in Fig 3. The SVM showed the highest accuracy (0.717, 95% CI, 0.681–0.752).

**Table 3:** Performance of the machine learning and Apfel model.

Model	AUROC	Accuracy	Sensitivity	Specificity
	(95% CI)	(95% CI)	(95% CI)	(95% CI)
LR	0.576	0.544	0.88	0.20
	(0.520–0.633)	(0.504–0.582)	(0.84–0.91)	(0.15–0.24)
KNN	0.597	0.649	0.87	0.20
	(0.537–0.656)	(0.611–0.686)	(0.83–0.90)	(0.15–0.26)
DT	0.561	0.550	0.86	0.17
	(0.498–0.625)	(0.510–0.589)	(0.82–0.89)	(0.13–0.22)
RF	0.610	0.652	0.88	0.22
	(0.552–0.668)	(0.614–0.689)	(0.84–0.91)	(0.17–0.28)
GBM	0.580	0.602	0.86	0.19
	(0.520–0.639)	(0.564–0.640)	(0.83–0.90)	(0.14–0.24)
SVM	0.649	0.717	0.88	0.25
	(0.592–0.707)	(0.681–0.752)	(0.84–0.90)	(0.19–0.33)
**ANN**	**0.686**	0.593	0.92	0.24
	**(0.630–0.742)**	(0.554–0.631)	(0.89–0.95)	(0.20–0.29)
Apfel	0.643	0.523	0.92	0.21
	(0.596–0.690)	(0.484–0.562)	(0.88–0.95)	(0.17–0.26)

AUROC, area under the receiver operating characteristic; LR, logistic regression; KNN, k-nearest neighbors; DT decision tree; RF, random forest; GBM, gradient boosting machine; SVM, support vector machine; ANN, artificial neural networks

The entire code used in this study is available online without restrictions (https://github.com/jgshim/IV-PCA). The detailed hyperparameters of the machine learning model used in this study can be found in the S1 Table.

## Discussion

We analyzed and compared the predictive ability of seven machine learning approaches and the Apfel model to predict PONV during 24 h after surgery. The results showed that the ANN method had the largest AUROC for identifying PONV using clinical data. The key findings were as follows: (1) machine learning models such as ANN and SVM showed better performance than the Apfel model and (2) feature selection using recursive feature elimination improved human insight into complex and non-linear models associated with PONV. To our knowledge, this is the first study to predict the occurrence of PONV by comparing various classification machine learning approaches with the Apfel model.

Conventional machine learning approaches generally work efficiently with traditional datasets and allow for nonlinear relationships between predictors but may deteriorate with high-dimensional problems [24]. We explored the number of selected features using the wrapped algorithm used in the recursive feature elimination procedure. By means of dimensionality reduction, the dependencies and collinearity that may exist in the model can be eliminated to improve performance.

Although volatile anesthetics was an important factor of PONV in a previous study [25], in this study, the type of volatile anesthetics or intravenous anesthesia was not helpful in predicting PONV. An increase in the duration of anesthesia was associated with a reduction in PONV, which is inconsistent with previous results [26]. The increase in PONV with the use of preintubation opioids or laparoscopic surgery is evidenced in previous studies [27, 28]. Hypotension occurring during shoulder surgery may be a major factor of PONV and was used as one of the features in our study [12, 29].

We believe that anesthesiologists in the operating room can help manage PONV. For example, considering the possibility of PONV, the ANN or SVM model could be useful in deciding whether to take preemptive measures, such as preparing an antiemetic in advance or continuing follow-up and observation. Furthermore, cost-effective management will be possible because the models require only 13 clinical variables to identify patients at a high risk of PONV.

The patients with chemotherapy history are at high risk for opioid induced PONV [30]. However, the number of cases of postchemotherapy patients were very small. Even if they had a history of cancer, it was not clear whether they had received chemotherapy. Thus, we were not able to include the postchemotherapy patient group as the input variable. Including chemotherapy as an input factor in further studies may improve the performance of the PONV prediction model.

It is clear that PONV is a distressing side effect in patients. A suitable screening test for PONV should include adequate sensitivity and specificity, and be acceptable to both patient and medical practitioners. Having high sensitivity but low specificity may lead to inappropriate preemptive measures. For instance, a patient with a low risk of PONV might be given anti-emetics. Therefore, careful attention should be paid when used as a screening tool.

There are some limitations to our study. First, we are not sure that the amount of data we used was enough to work on machine-learning problems, considering the complexity of the problem and nonlinear algorithms. Further data about PONV should be collected to improve the predictive power. Second, one of the PCA teams in the anesthesiology department visited only once during the day after surgery and asked about the effects and complications of PCA. As a result, because of recall bias, the PONV occurrence rate may have been underestimated. Third, because our study analyzed data from a single center, it might not be possible to apply our model to a wider population. Further studies are needed, with large heterogeneous samples, to improve generalizability.

## Conclusions

In summary, we developed and compared various machine learning models and the Apfel model to predict the occurrence of PONV using IV-PCA. We expect our results to help reduce PONV by helping clinicians predict it and take preemptive actions.

## Supporting information

S1 Table Optimal hyperparameters of all machine learning models. (DOCX)

## Abbreviations

ANN: Artificial neural network
AUROC: Area under the receiver operating characteristic curve
CI: Confidence interval
DT: Decision tree
GBM: Gradient boosting machine
KNN: K-nearest neighbors
PCA: Patient-controlled analgesia
PONV: Postoperative nausea and vomiting
RF: Random forest
SVM: Support vector machine
TIVA: Total intravenous anesthesia

## References

[1] Choi DH, Ko JS, Ahn HJ, Kim JA. A korean predictive model for postoperative nausea and vomiting. J Korean Med Sci. 2005;20(5):811–5. Epub 2005/10/15. doi: 10.3346/jkms.2005.20.5.811 ; PubMed Central PMCID: PMC2779278. 16224155 PMC2779278

[2] Gan TJ, Belani KG, Bergese S, Chung F, Diemunsch P, Habib AS, et al. Fourth Consensus Guidelines for the Management of Postoperative Nausea and Vomiting. Anesth Analg. 2020;131(2):411–48. Epub 2020/05/30. doi: 10.1213/ANE.0000000000004833 . 32467512

[3] Lee MJ, Lee KC, Kim HY, Lee WS, Seo WJ, Lee C. Comparison of ramosetron plus dexamethasone with ramosetron alone on postoperative nausea, vomiting, shivering and pain after thyroid surgery. Korean J Pain. 2015;28(1):39–44. Epub 2015/01/16. doi: 10.3344/kjp.2015.28.1.39 ; PubMed Central PMCID: PMC4293505. 25589945 PMC4293505

[4] de Boer HD, Detriche O, Forget P. Opioid-related side effects: Postoperative ileus, urinary retention, nausea and vomiting, and shivering. A review of the literature. Best Pract Res Clin Anaesthesiol. 2017;31(4):499–504. Epub 2018/05/10. doi: 10.1016/j.bpa.2017.07.002 . 29739538

[5] Choi JB, Shim YH, Lee YW, Lee JS, Choi JR, Chang CH. Incidence and risk factors of postoperative nausea and vomiting in patients with fentanyl-based intravenous patient-controlled analgesia and single antiemetic prophylaxis. Yonsei Med J. 2014;55(5):1430–5. Epub 2014/07/23. doi: 10.3349/ymj.2014.55.5.1430 ; PubMed Central PMCID: PMC4108834. 25048507 PMC4108834

[6] Grass JA. Patient-controlled analgesia. Anesth Analg. 2005;101(5 Suppl):S44–61. Epub 2005/12/13. doi: 10.1213/01.ANE.0000177102.11682.20 . 16334492

[7] McNicol ED, Ferguson MC, Hudcova J. Patient controlled opioid analgesia versus non-patient controlled opioid analgesia for postoperative pain. Cochrane Database Syst Rev. 2015;2015(6):Cd003348. Epub 2015/06/03. doi: 10.1002/14651858.CD003348.pub3 ; PubMed Central PMCID: PMC7387354 interest to declare JH has no relevant conflicts of interest to declare. 26035341 PMC7387354

[8] Apfel CC, Läärä E, Koivuranta M, Greim CA, Roewer N. A simplified risk score for predicting postoperative nausea and vomiting: conclusions from cross-validations between two centers. Anesthesiology. 1999;91(3):693–700. Epub 1999/09/15. doi: 10.1097/00000542-199909000-00022 . 10485781

[9] Weilbach C, Rahe-meyer N, Raymondos K, Weissig A, Scheinichen D, Piepenbrock S. Postoperative nausea and vomiting (PONV): usefulness of the Apfel-score for identification of high risk patients for PONV. Acta Anaesthesiol Belg. 2006;57(4):361–3. Epub 2007/01/24. . 17236637

[10] Engel JM, Junger A, Hartmann B, Little S, Schnöbel R, Mann V, et al. Performance and customization of 4 prognostic models for postoperative onset of nausea and vomiting in ear, nose, and throat surgery. J Clin Anesth. 2006;18(4):256–63. Epub 2006/06/27. doi: 10.1016/j.jclinane.2005.10.003 . 16797426

[11] van den Bosch JE, Kalkman CJ, Vergouwe Y, Van Klei WA, Bonsel GJ, Grobbee DE, et al. Assessing the applicability of scoring systems for predicting postoperative nausea and vomiting. Anaesthesia. 2005;60(4):323–31. Epub 2005/03/16. doi: 10.1111/j.1365-2044.2005.04121.x . 15766334

[12] Chae D, Kim SY, Song Y, Baek W, Shin H, Park K, et al. Dynamic predictive model for postoperative nausea and vomiting for intravenous fentanyl patient-controlled analgesia. Anaesthesia. 2020;75(2):218–26. Epub 2019/09/19. doi: 10.1111/anae.14849 . 31531854

[13] Wu HY, Gong CA, Lin SP, Chang KY, Tsou MY, Ting CK. Predicting postoperative vomiting among orthopedic patients receiving patient-controlled epidural analgesia using SVM and LR. Sci Rep. 2016;6:27041. Epub 2016/06/02. doi: 10.1038/srep27041 ; PubMed Central PMCID: PMC4887988. 27247165 PMC4887988

[14] Gong CS, Yu L, Ting CK, Tsou MY, Chang KY, Shen CL, et al. Predicting postoperative vomiting for orthopedic patients receiving patient-controlled epidural analgesia with the application of an artificial neural network. Biomed Res Int. 2014;2014:786418. Epub 2014/08/28. doi: 10.1155/2014/786418 ; PubMed Central PMCID: PMC4138736. 25162027 PMC4138736

[15] Yuh-Jyh H, Jia-Ying S, Tien-Hsiung K. Predicting Postoperative Nausea and Vomiting Under Patient-Controlled Analgesia Medication: A Study of Machine Learning Approaches. Prim Health Care. 2017;7(272):2167–1079.1000272.

[16] Lötsch J, Ultsch A. Machine learning in pain research. Pain. 2018;159(4):623–30. Epub 2017/12/02. doi: 10.1097/j.pain.0000000000001118 ; PubMed Central PMCID: PMC5895117 the end of this article. 29194126 PMC5895117

[17] Obermeyer Z, Emanuel EJ. Predicting the Future—Big Data, Machine Learning, and Clinical Medicine. N Engl J Med. 2016;375(13):1216–9. Epub 2016/09/30. doi: 10.1056/NEJMp1606181 ; PubMed Central PMCID: PMC5070532. 27682033 PMC5070532

[18] Wang Y, Lei L, Ji M, Tong J, Zhou CM, Yang JJ. Predicting postoperative delirium after microvascular decompression surgery with machine learning. J Clin Anesth. 2020;66:109896. Epub 2020/06/07. doi: 10.1016/j.jclinane.2020.109896 . 32504969

[19] Panesar SS, D’Souza RN, Yeh FC, Fernandez-Miranda JC. Machine Learning Versus Logistic Regression Methods for 2-Year Mortality Prognostication in a Small, Heterogeneous Glioma Database. World Neurosurg X. 2019;2:100012. Epub 2019/06/21. doi: 10.1016/j.wnsx.2019.100012 ; PubMed Central PMCID: PMC6581022. 31218287 PMC6581022

[20] Kendale S, Kulkarni P, Rosenberg AD, Wang J. Supervised Machine-learning Predictive Analytics for Prediction of Postinduction Hypotension. Anesthesiology. 2018;129(4):675–88. Epub 2018/08/04. doi: 10.1097/ALN.0000000000002374 . 30074930

[21] Lee HC, Yoon SB, Yang SM, Kim WH, Ryu HG, Jung CW, et al. Prediction of Acute Kidney Injury after Liver Transplantation: Machine Learning Approaches vs. Logistic Regression Model. J Clin Med. 2018;7(11). Epub 2018/11/11. doi: 10.3390/jcm7110428 ; PubMed Central PMCID: PMC6262324. 30413107 PMC6262324

[22] Beretta L, Santaniello A. Nearest neighbor imputation algorithms: a critical evaluation. BMC Med Inform Decis Mak. 2016;16 Suppl 3(Suppl 3):74. Epub 2016/07/28. doi: 10.1186/s12911-016-0318-z ; PubMed Central PMCID: PMC4959387. 27454392 PMC4959387

[23] Wang Q, Luo Z, Huang J, Feng Y, Liu Z. A Novel Ensemble Method for Imbalanced Data Learning: Bagging of Extrapolation-SMOTE SVM. Comput Intell Neurosci. 2017;2017:1827016. Epub 2017/03/03. doi: 10.1155/2017/1827016 ; PubMed Central PMCID: PMC5304315 of this paper. 28250765 PMC5304315

[24] Darst BF, Malecki KC, Engelman CD. Using recursive feature elimination in random forest to account for correlated variables in high dimensional data. BMC Genet. 2018;19(Suppl 1):65. Epub 2018/09/27. doi: 10.1186/s12863-018-0633-8 ; PubMed Central PMCID: PMC6157185. 30255764 PMC6157185

[25] Apfel CC, Kranke P, Katz MH, Goepfert C, Papenfuss T, Rauch S, et al. Volatile anaesthetics may be the main cause of early but not delayed postoperative vomiting: a randomized controlled trial of factorial design. Br J Anaesth. 2002;88(5):659–68. Epub 2002/06/18. doi: 10.1093/bja/88.5.659 . 12067003

[26] SINCLAIR CHUNG DR F, MEZEI G. Can Postoperative Nausea and Vomiting Be Predicted? Survey of Anesthesiology. 2000;44(1):3–4. 10.1097/00000542-199907000-00018 10422935

[27] Roberts GW, Bekker TB, Carlsen HH, Moffatt CH, Slattery PJ, McClure AF. Postoperative nausea and vomiting are strongly influenced by postoperative opioid use in a dose-related manner. Anesth Analg. 2005;101(5):1343–8. Epub 2005/10/26. doi: 10.1213/01.ANE.0000180204.64588.EC . 16243992

[28] Cao X, White PF, Ma H. An update on the management of postoperative nausea and vomiting. J Anesth. 2017;31(4):617–26. Epub 2017/04/30. doi: 10.1007/s00540-017-2363-x . 28455599

[29] Pusch F, Berger A, Wildling E, Tiefenthaler W, Krafft P. The effects of systolic arterial blood pressure variations on postoperative nausea and vomiting. Anesth Analg. 2002;94(6):1652–5, table of contents. Epub 2002/05/29. doi: 10.1097/00000539-200206000-00054 . 12032046

[30] Kumar A, Solanki SL, Gangakhedkar GR, Shylasree TS, Sharma KS. Comparison of palonosetron and dexamethasone with ondansetron and dexamethasone for postoperative nausea and vomiting in postchemotherapy ovarian cancer surgeries requiring opioid-based patient-controlled analgesia: A randomised, double-blind, active controlled study. Indian J Anaesth. 2018;62(10):773–9. Epub 2018/11/18. doi: 10.4103/ija.IJA_437_18 ; PubMed Central PMCID: PMC6190431. 30443060 PMC6190431

