# Supplementary material for: Machine learning for prediction of postoperative nausea and vomiting in patients with intravenous patient-controlled analgesia
Source: PLoS One. 2022 Dec 22;17(12):e0277957. doi: 10.1371/journal.pone.0277957 (PMC9778492; doi:10.1371/journal.pone.0277957)
Supplement: S1 Table — (DOCX) [file pone.0277957.s001.docx]

S1 Table. Optimal hyperparameters of all machine learning models

| Model | Optimal hyperparameters |
| --- | --- |
| LR | nIter* = 31 |
| KNN | k = 8 |
| DT | Maximum depth = 5  Criterion = Gini index |
| RF | mtry** = 3 |
| GBM | Maximum depth = 3  Number of estimators = 50,  Gamma = 0 |
| SVM | degree = 3, scale = 0.1 and C = 1.0 |
| ANN | Number of hidden layers = 2  Number of nodes in a layer = 32, 16 |

LR, logistic regression; KNN, k-nearest neighbors; DT, decision tree; RF, random forest; GBM, gradient boosting machine; SVM, support vector machine; ANN, artificial neural networks

*nIter indicates an integer, describing the number of iterations for which boosting should be run.

**mtry indicates the number of variables available for splitting at each tree node.
